# Establishing an imaging protocol for pediatric trauma in a rural hospital

**DOI:** 10.1016/j.sipas.2023.100186

**Published:** 2023-06-09

**Authors:** Vrutant Patel, Rahima Khatun, Mary Carmack, Jeanet Calhoun, Joon K. Shim

**Affiliations:** a Department of Surgery, Bassett Medical Center, Cooperstown, NY, USA; b Columbia University Vagelos College of Physicians and Surgeons, New York, NY, USA

**Keywords:** Pediatric trauma, Trauma imaging, Imaging guidelines, Radiation exposure, Rural trauma center

## Abstract

**Background:**

Rural hospitals cover 20% of the United States (US) population with only 10% of physician coverage. A mismatch exists in pediatric trauma resources as there is overwhelming trauma support concentrated in urban trauma centers. Well-established guidelines for evaluating pediatric trauma patients in resource-limited environments are currently not available. Herein we identify the imaging practices at a level III rural trauma center to establish a protocol for handling pediatric traumas.

**Materials and Methods:**

The National Trauma Data Bank was used to identify 155 pediatric trauma patients (age <17 years) between 2017 and 2021. A single-center retrospective chart review was performed to identify patient demographics, mechanism of injury, imaging performed and pertinent imaging findings, and management of the patient i.e., whether they were discharged, admitted, or transferred.

**Results:**

Blunt mechanisms were responsible for most traumas (90%). There were 64 patients (41.3%) who received imaging. Falls (49.3%) were the most common injury. Most of the patients were discharged home (73.4%) and 23.9% were transferred to a tertiary center. The mean time for transfer to a tertiary center was ∼176 min. The most frequently performed type of surgical intervention was orthopedic (59.3%).

**Conclusion:**

An established pediatric trauma imaging protocol is warranted to adopt a higher level of pediatric trauma care for treatment and/or stabilization purposes. Using a tertiary care model and established pediatric trauma guidelines, we propose a model for use in resource-limited rural settings and aim to reduce unnecessary imaging of pediatric trauma patients and overall radiation exposure.

## Introduction

Pediatric trauma is the leading cause of death for children in the United States (US) [1]. There are only approximately 35 Level I pediatric trauma centers and 32 Level II centers in the US as of 2012 [2]. Rural communities are known to be underserved. Almost 20% of the country's population lives in rural areas, but less than 10% of physicians practice in these communities [3]. With regards to trauma centers, there is a major inequity in access to trauma centers across the US with level III to level IV trauma centers predominantly situated in rural and underserved areas and higher-level centers located in metropolitan areas [2].

Although there are well-established guidelines for evaluating pediatric trauma patients in large trauma centers, guidelines adapted specifically for the unique challenges of rural centers are lacking [4]. A mismatch exists in pediatric trauma resources in the United States. Limited availability of pediatric specialists, extended transit time and distance to higher levels of care, and limited emergency department resources are challenges in the management of pediatric trauma cases. Computer tomography (CT), while a resourceful imaging tool, carries a significant radiation burden on the pediatric population. There is an increasing trend noted in the utilization of CT scans for pediatric trauma which in turn has increased the rate of malignancy related to radiation exposure [5].

Our institution is a healthcare network covering a vast geographical area in central New York, in the northernmost part of the Appalachian region. Like many rural areas, there are high rates of poverty and limited public transportation options. Our facility is a 180-bed acute care teaching facility in rural upstate New York. Patients often present at our smaller satellite healthcare centers for an initial assessment before they are transferred to our facility. The pediatric traumas evaluated at our facility are triaged by adult general surgery residents and surgeons, with familiarity more with the adult population rather than pediatric. A standardized protocol adopted across our healthcare network would help optimize care for pediatric trauma patients.

In this study, we identify imaging practices at our rural Level III trauma center in the evaluation of pediatric trauma patients. We have developed a protocol for handling pediatric traumas to standardize care within our own facility, as well as to provide one for similar rural facilities. There are promising studies that have documented algorithm-based triaging and institute-wide teaching that can lead to a reduction in radiation exposure [6]. We aim to characterize and evaluate the current use of CT scans in pediatric trauma patients with the hopes of optimizing the imaging protocol.

## Methods

We used the National Trauma Data Bank (NTDB) to select patients for this study. All trauma patients seen in our Emergency Departments (ED) and their associated data are submitted to the NTDB. We used the inclusion criteria of patients 17 years old and younger who were evaluated in our ED from 2017 through 2021. The Institutional Review Board waiver for consent was obtained as this was a retrospective quality improvement study.

We performed a single-center retrospective chart review to identify patient demographics, mechanism of injury, type of imaging performed, pertinent imaging findings, and discharge disposition. Patients presenting to the ED were triaged based on the following categories: trauma alert (activation as level 1 or 2 depending on the injury), trauma evaluation (originally did not meet the criteria for activation but needed surgical service assistance), and trauma consult (patient is at an outside facility and transferred to main campus). The primary endpoint was to evaluate the type of radiological studies being performed i.e., CT scans. This data is aimed to provide insight into imaging patterns at our facility and allow for data comparison after the implementation of the proposed protocol.

The imaging protocol was formed using the existing guidelines such as Pediatric Emergency Care Applied Research Network (PECARN) for determining the need for imaging studies at the time of presentation [7,8]. This protocol was further refined by obtaining guidance from faculty at a Level 1 pediatric trauma center.

## Results

A total of 155 patients were included in our study population. The majority of our patients were male (61.3%) with an average age and body mass index (BMI) of 9.2 years and 22.4, respectively [Table 1]. Between trauma activation and evaluations, 95.5%, had presented to the ED (*n* = 148). Seven patients (4.5%) were required to transfer from an affiliated hospital within the network for consultation to the main hospital.

**Table 1:** Characteristics of study population, total (*n*) = 155 patients.

	***n* (%)**
**Sex**	
Male	95 (61.3%)
Female	60 (38.7%)
**Age, mean**	9.2 years
**BMI, mean**	22.4
**Trauma Level**	
Level 1	6 (3.9%)
Level 2	30 (19.4%)
Trauma Evaluation	112 (72.2%)
Consult	7 (4.5%)
**Mechanism of Injury**	
Blunt	140 (90.3%)
Penetrating	15 (9.7%)

**Fig. 1:**
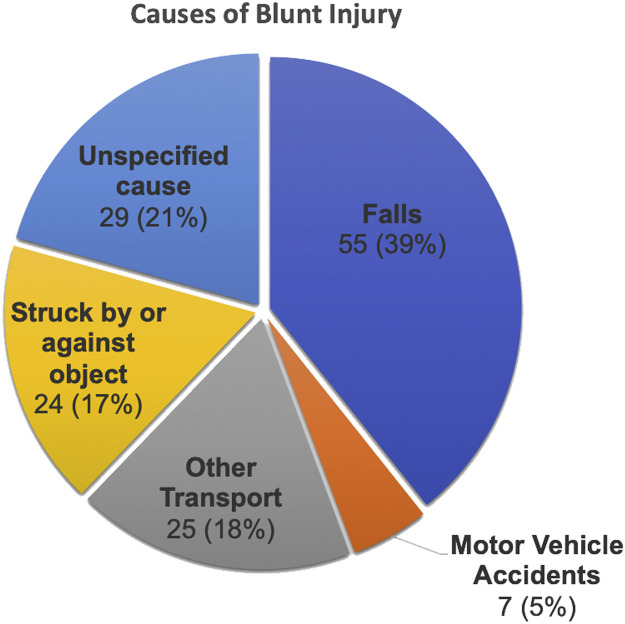
Breakdown of blunt injuries identified on trauma assessment.

The predominant mechanism of trauma was blunt injury accounting for 90.3% and penetrating injury was 9.7% [Table 1]. Blunt traumas were further characterized into different mechanisms which showed falls accounted for most injuries, 39%, followed by unspecified causes (21%) and struck by or against an object (17%) [Fig. 1]. Only one penetrating injury required formal exploration and repair of injury in the operating room. Otherwise, the remaining were managed with local wound care. The most frequent surgical intervention provided was orthopedics, 59.3%. The decision to take the patient to the operative room was usually based on x-ray imaging of the involved injury.

For the 155 patients, 192 CT scans were obtained. CT head was the most common imaging performed (36.1%) during the evaluation, followed by CT cervical spine (23.9%) [Table 2]. Furthermore, 65 out of 155 patients (42%) received some CT imaging. All patients had received some X-ray imaging, the most common being the chest X-ray. Thirty-six patients (23.2%) required transfer to a higher level of care due to the complex nature of their injuries, whereas 116 patients (74.8%) were discharged home with instructions to return to the ED if needed [Table 2]. All the patients who were transferred to a higher level of care received CT imaging whereas 50% of the patients who were discharged to home received CT imaging. The average transfer time from the presentation to the tertiary center was approximately 176 min. The most common reason for the transfer was head injury requiring a higher level of care.

**Table 2:** Imaging performed, and discharge disposition.

CT Scans (*n* = 192)	
CT head without contrast	56 (36.1%)
CT cervical spine without contrast	37 (23.9%)
CT abdomen pelvis with IV contrast (without oral)	28 (18.0%)
CT chest with contrast	25 (16.1%)
CT thoracic spine with contrast	17 (10.9%)
CT sinus face without contrast	15 (9.7%)
CT lumbar spine with contrast	14 (9.0%)
**Discharge Disposition**	
Home	116 (74.8%)
Transfer to higher level of care	36 (23.2%)
Other * Jail = 2, Death = 1	3 (2.0%)

## Discussion

Our institute is an adult level III trauma center and has seen similar trends compare to previous studies. Pediatric traumas are primarily triaged by the adult surgical team. Forty-two percent of patients triaged at our facility had undergone CT imaging, which compared to pediatric centers (21–23%) is significantly high [9]. The imaging obtained at the institute is mainly provider dependent as there is no protocol exists. The concept of as low as reasonably achievable or ALARA is being widely accepted when it comes to pediatric radiology protocol. Originally intended for pediatric oncology, this concept is now being applied to trauma imaging as well [10].

Pediatric trauma remains a leading cause of mortality in children [1]. Recent studies have shown that children triaged at a pediatric trauma center have a higher likelihood of improved mortality while undergoing less radiation during evaluation [11,12]. Blunt traumas are the most concerning injuries as these injuries tend to hide underlying pathology, which makes them difficult to diagnose. One of the primary reasons for an increase in CT imaging in blunt traumas is attributed to the uncertainty of injury profile. Studies have shown that pediatric trauma patients managed at adult trauma centers were 1.8 times more likely to receive whole-body CT (WBCT) imaging than patients in pediatric trauma centers with no difference in mortality rate [12].

Established guidelines exist such as Pediatric Emergency Care Applied Research Network (PECARN) which aid in identifying patients with specific injuries who require CT imaging. However, in rural hospitals with limited resources, implementation of such guidelines can become cumbersome. Having a standardized protocol though such as the one we proposed based on the concept of ALARA and an institution's capabilities can reduce the imaging rate, as well as associated exposure risk and costs.

**Fig. 2:**
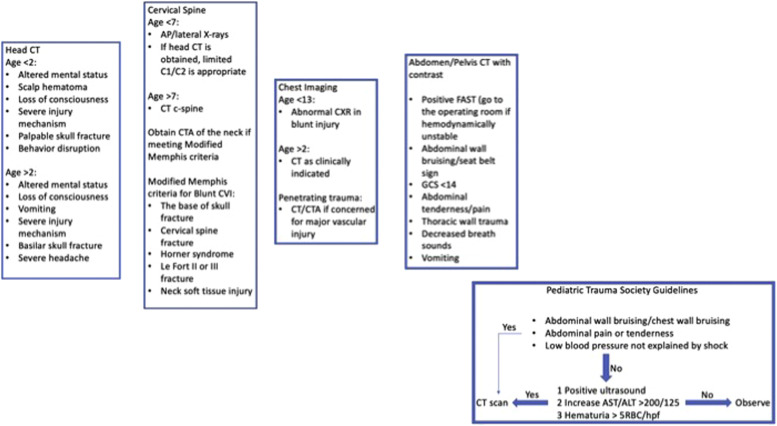
Proposed Imaging Guidelines for Evaluation of Pediatric Traumas.

This quality-improvement project is not without limitations. Given the limited number of pediatric trauma patients seen at our institution, our sample size is small. Furthermore, this was a retrospective chart review and as such, there may be inconsistencies with charting. Lastly, this protocol has not yet been validated. However, our goal for the next step is to implement this protocol to validate whether it has led to more informed decision-making regarding pediatric trauma assessment. Furthermore, the protocol shown in Fig. 2 is planned to be released across the hospital network and data collection for prospective study will take place. Data will then be compared at 2, 5, and 7-year intervals. This will help to identify the trend and further make changes at each interval.

Non-specialized centers will be required to have a higher level of basic care to stabilize pediatric patients to compensate for geographic and transfer-related delays in care. Unnecessary imaging is just another source of delay to transfer and definitive treatment. With limited pediatric trauma centers in the country, most of the initial trauma assessment occurs at smaller healthcare centers like our own, where initial assessment may be a chance to optimize care.

## Conclusion

Our level three rural trauma center utilizes an adult trauma team for pediatric trauma activations. Studies have shown that the use of CT scans has been significantly higher at adult trauma centers in comparison to pediatric trauma centers. An established pediatric trauma imaging protocol is warranted as rural hospitals will be required to adopt a higher level of pediatric trauma care for treatment and/or stabilization purposes. Using a tertiary care model and established pediatric trauma guidelines, we propose a model for use in resource-limited rural settings as seen in Fig. 2. With this guideline, we aim to reduce the overall imaging obtained for pediatric trauma patients as well as radiation exposure at a resource-limited rural hospital.

## CRediT authorship contribution statement

**Vrutant Patel:** Conceptualization, Data curation, Formal analysis, Investigation, Methodology, Supervision, Validation, Visualization, Writing – original draft, Writing – review & editing. **Rahima Khatun:** Data curation, Formal analysis, Investigation, Methodology, Writing – original draft, Writing – review & editing. **Mary Carmack:** Methodology, Investigation, Data curation, Writing – original draft. **Jeanet Calhoun:** Methodology, Investigation, Data curation, Writing – original draft, Writing – review & editing. **Joon K. Shim:** Conceptualization, Data curation, Formal analysis, Investigation, Methodology, Project administration, Supervision, Validation, Visualization, Writing – original draft, Writing – review & editing.

## Declaration of Competing Interest

The authors declare that they have no known competing financial interests or personal relationships that could have appeared to influence the work reported in this paper.
